# Efficient reconstruction of cell lineage trees for cell ancestry and cancer

**DOI:** 10.1093/nar/gkad254

**Published:** 2023-04-07

**Authors:** Yeongjun Jang, Liana Fasching, Taejeong Bae, Livia Tomasini, Jeremy Schreiner, Anna Szekely, Thomas V Fernandez, James F Leckman, Flora M Vaccarino, Alexej Abyzov

**Affiliations:** Department of Quantitative Health Sciences, Center for Individualized Medicine, Mayo Clinic, Rochester, MN 55905, USA; Child Study Center, Yale University, New Haven, CT 06520, USA; Department of Quantitative Health Sciences, Center for Individualized Medicine, Mayo Clinic, Rochester, MN 55905, USA; Child Study Center, Yale University, New Haven, CT 06520, USA; Child Study Center, Yale University, New Haven, CT 06520, USA; Department of Neurology, Yale University, New Haven, CT 06520, USA; Child Study Center, Yale University, New Haven, CT 06520, USA; Department of Psychiatry, Yale University, New Haven, CT 06511, USA; Child Study Center, Yale University, New Haven, CT 06520, USA; Child Study Center, Yale University, New Haven, CT 06520, USA; Department of Neuroscience, Yale University, New Haven, CT 06520, USA; Yale Kavli Institute for Neuroscience, New Haven, CT 06520, USA; Department of Quantitative Health Sciences, Center for Individualized Medicine, Mayo Clinic, Rochester, MN 55905, USA

## Abstract

Mosaic mutations can be used to track cell ancestries and reconstruct high-resolution lineage trees during cancer progression and during development, starting from the first cell divisions of the zygote. However, this approach requires sampling and analyzing the genomes of multiple cells, which can be redundant in lineage representation, limiting the scalability of the approach. We describe a strategy for cost- and time-efficient lineage reconstruction using clonal induced pluripotent stem cell lines from human skin fibroblasts. The approach leverages shallow sequencing coverage to assess the clonality of the lines, clusters redundant lines and sums their coverage to accurately discover mutations in the corresponding lineages. Only a fraction of lines needs to be sequenced to high coverage. We demonstrate the effectiveness of this approach for reconstructing lineage trees during development and in hematologic malignancies. We discuss and propose an optimal experimental design for reconstructing lineage trees.

## Graphical Abstract

**Graphical Abstract:**
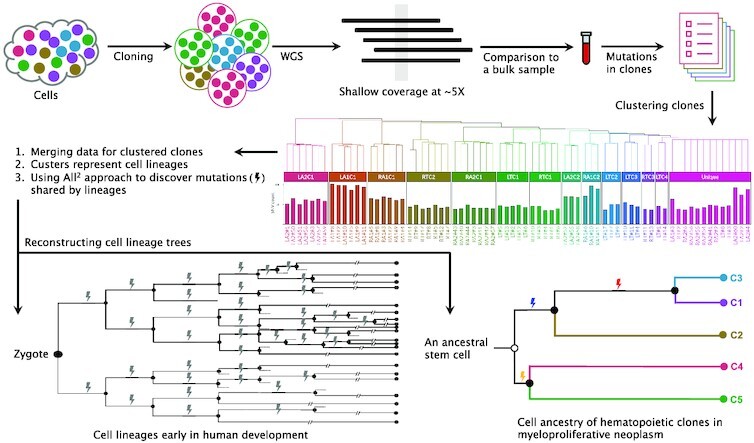
Efficient reconstruction of cell lineage trees for cell ancestry and cancer.

## INTRODUCTION

Understanding the structure and dynamics of the human cell lineage tree in development and aging is a central question in medicine and biology. Understanding cell lineages in cancer development is also important for studying dynamics of cancer progression and metastasis. To map cellular ancestry in humans, recent studies typically collected biopsies from living or post-mortem individuals, sampled single cells, cloned them via different approaches, sequenced each clone and cross-compared the sequence across clones to discover mosaic mutations in the founder cell of each clone (1–6). Mutation sharing across multiple cell clones allowed reconstructing branches in the cell ancestry tree, which represent individual cell divisions during early development starting from the zygote. Deeper trees with more completely reconstructed branches allow studying later stages of development and in greater detail. The major factor affecting tree depth (i.e. how many branches can be reconstructed) is the number of single cells analyzed. We estimate that ∼3000 cells are needed to completely reconstruct lineage trees reflecting the time of gastrulation (when the human embryo is ∼600–1000 cells in size), as sampled cells are redundant in lineage representation (i.e. multiple cells can sample the same early lineage). Furthermore, due to local clonal expansion in a tissue or organ, many cells from the same biopsy will carry almost the same set of mutations and therefore will not be useful to discriminate branches of the ancestry tree. Even with sequencing costs having dropped dramatically in recent years, carrying out extensive sampling and analyses of clones’ genomes is cost prohibitive for the wide application of lineage analyses in the population.

To mitigate the cost, some studies used shallower (15×) instead of standard (30×) sequencing coverage of clones (3,6). In our previous study, we devised a strategy where we prescreened clones for redundancy (1). Specifically, we performed multiple skin biopsies in two living individuals and derived clonal induced pluripotent stem cell (iPSC) lines from cultured fibroblasts of each biopsy. We then did the following: (i) derived a draft lineage tree from the sequencing and analysis of a few clones; (ii) defined marker mutations for early lineages; (iii) genotyped early mutations in all remaining lines; and (iv) selected and sequenced only the nonredundant lines. While such a screening strategy led us to sequence only about a quarter of all available lines, it was a time-consuming effort. Here, we describe a cost-effective and swift strategy for mosaic mutation discovery and lineage reconstruction using shallow (5×)-coverage sequencing of all available lines/clones and demonstrate its power using the fibroblast-derived iPSC lines in our previous study. The core idea is that, since redundant iPSC lines or clones (as in cancers) share all or most mutations, a sufficient count of mutations shared by the lines can be discovered from shallow sequencing. Based on the shared mutations, redundant lines/clones can then be identified and clustered. Data for shallow sequenced lines in each cluster can be combined to yield higher coverage of the corresponding lineage, allowing for comprehensive discovery of mosaic mutations and thereby enabling accurate reconstruction of lineage trees.

## MATERIALS AND METHODS

### Downsampling of high-coverage iPSC lines

For downsampling BAM files of 25 iPSC lines with high coverage that were aligned to the human reference genome hg19, we used SAMtools (7) with the following commands and parameters:

samtools view -@ 8 -s $(samtools stats -@ 8 high_coverage_line.bam chr22 |grep ^COV |awk ‘{reads+ = $4; coverages+ = ($3*$4)} END {avg = coverages/reads; print 42 + 5/avg}’) -b high_coverage_line.bam > down_sampled_line.bam && samtools index -@ 8 down_sampled_line.bam

Coverages of high-coverage data varied from 30× to >60× (Supplementary Figure S1A). To get BAM files downsampled exactly to 5× average (Supplementary Figure S1B), we applied the different sampling probability to each line according to their average coverage in the original data. Coverage distribution of downsampled BAM files perfectly matched with those of shallow-coverage data that were originally sequenced at 5× coverage (Supplementary Figure S1C).

### Calling mutations in iPSC lines relative to blood

In addition to 25 downsampled lines, all reads of 47 lines originally sequenced at shallow coverage (5×) were mapped to the human reference genome hg19. The duplicated reads were marked and removed by Picard (available at http://broadinstitute.github.io/picard), and indel realignment and base quality score recalibration were performed with GATK (8). Somatic single-nucleotide variants (SNVs) were called using Mutect2 from GATK version 4.1.8 in each of the 72 shallow-coverage lines against the genomic DNA of blood cells from the same individual. To ensure the high confidence of called SNV sets, we applied the following two filters: (i) mutation calls in inaccessible genomic regions according to the 1000 Genomes mappability mask were eliminated and (ii) mutation calls were required to be supported by at least three reads.

### Clustering of iPSC lines using mutations in nuclear genome

**Figure 1.**
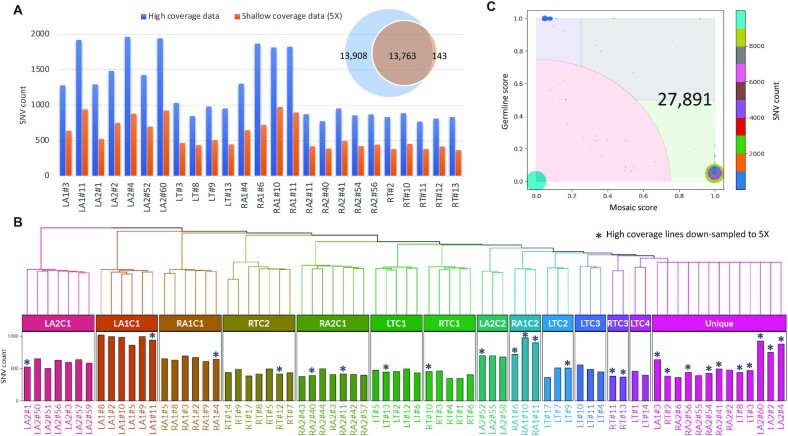
Hierarchical clustering of iPSC lines from shallow-coverage whole-genome sequencing (WGS) data. (**A**) Comparison of counts of mosaic SNVs discovered from the original high coverage (>30×) and from downsampling original data to 5×. Bar plot compares counts per iPSC line. Venn diagram compares total counts. iPSC lines are named using the regions where they are from (LA, left arm; RA, right arm; LT, left thigh; RT, right thigh), followed by biopsy number and the index of a line. (**B**) Hierarchical clustering of all iPSC lines based on shared SNVs discovered from 5× data. Lines previously sequenced at high coverage and downsampled are marked by *. Bar plot at the bottom shows counts of discovered mutations. (**C**) Distribution of scores for mutation calls from comprehensive pairwise comparison of clusters LA2C1 through LTC4 and high-coverage iPSC lines from the ‘Unique’ cluster. Each dot is a call for a mutation. Likely mosaic mutations (27891) are located in the green and gray areas (the gray area corresponds to high-frequency mosaic mutations), likely germline variants are in blue area and false calls are in red area.

To identify clusters for the 72 iPSC lines from shallow-coverage datasets, we applied a hierarchical clustering algorithm using SNVs that were called relative to blood in each line. First, for all iPSC lines we constructed the pairwise distance matrix using the distance measure 1 – *J*, where *J* is the Jaccard index calculated as the fraction of mutations called in both considered samples relative to those called in at least one sample. Ward’s minimum variance method was used for iteratively agglomerating clusters. At each step, the method merges a pair of clusters (or lines) that results in the minimum increase in total within-cluster distance variance after merging. We visualized the resulting clusters in a hierarchical tree with mutation counts in each line (Figure 1B) using the ‘ComplexHeatmap’ R package (9). In the dendrogram of the hierarchical tree, each leaf corresponds to one iPSC line. As we move up the tree, iPSC lines that have similar mutational events to each other are combined into a branch. The height of the branch along with the vertical axis indicates the dissimilarity between iPSC lines: the longer the height of the branch, the less similar the iPSC lines are. If there is an outlier cluster that combines iPSC lines with the longest height to the first branch and shows no hierarchical structure within the cluster, we define it as a ‘Unique’ cluster that includes multiple nonredundant iPSC lines representing unique lineages (Figure 1B).

We next estimated the minimum mutation burden in each line that should make our clustering approach work by simulating lower mutation burdens in each line. Simulating lower mutation burden was conducted by randomly downsampling sites with discovered mutations. Downsampling was conducted as follows: (i) mutation calls from all shallow-coverage iPSC lines were combined into the union call set; (ii) out of the set of union calls, 50%, 20%, 10% and 5% sites were randomly sampled; and (iii) in each iPSC line, only calls overlapping sampled sites were retained for clustering. While randomly downsampling to 50%, 20% and 10% mutation sites reproduced the same clusters as when using all discovered mutations, random downsampling to 5% (roughly 20–50 discovered mutations) resulted in some discrepancies in clustering, particularly for the ‘RA2C1’ cluster where iPSC lines had an overall lower mutation burden (Supplementary Figure S9). Consequently, we concluded that, when adjusted for sensitivity, a burden of ∼100 of mutations per line is sufficient for clustering redundant lines.

### Calling mutations for clusters and high-coverage iPSC lines

We used two strategies to generate BAM files from each cluster, which were used for identifying mosaic mutations across clusters and high-coverage iPSC lines. The first strategy is ‘sum all’, where (i) we summed all shallow-coverage lines in each cluster except the ‘Unique’ and (ii) among 13 iPSC lines in the ‘Unique’ cluster, 3 with shallow coverage (5×) were excluded and for other 10 sequenced at high coverage we utilized their full coverage. The second and slightly enhanced strategy is ‘pick one’, where (i) in seven clusters with >3 lines and except for the ‘Unique’ we merged data to get a BAM file with a coverage high enough to accurately discover mutations; (ii) we randomly picked a high-coverage line from four other small clusters that had 2–3 lines; (iii) we skipped the remaining two small clusters because they do not have any high-coverage line; and (iv) for lines in the ‘Unique’ cluster, the same strategy was applied as in the ‘sum all’.

**Figure 2.**
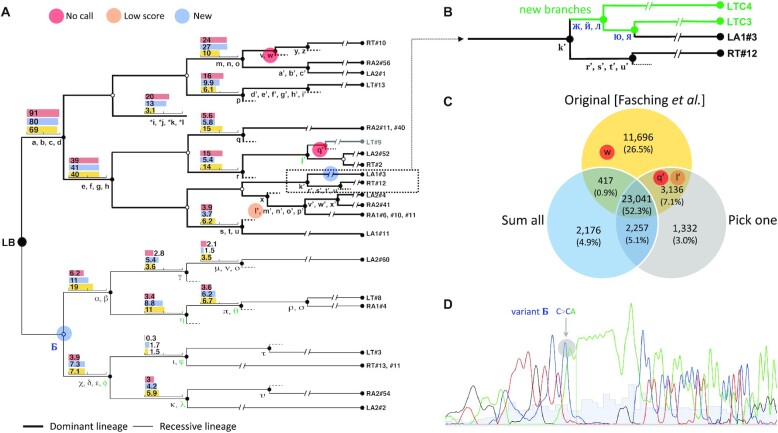
Reconstructing lineage tree from clustering iPSC lines and combining shallow-coverage WGS data. (**A**) Original lineage tree produced by Fasching *et al.* using high-coverage data (1) with designation of missing (red and orange circles) and new mutations (blue circles) when reconstructed from 5× data. Circles represent cells, with open circles marking cells with no created mutation, leading to ambiguous branching. Solid lines show likely parental relationships of lineages. iPSC lines are named at the terminal of the branches. Mosaic SNVs (black) and indels (green) are denoted by Latin and Greek letters. Four mutations (*i, *j, *k, *l) were discovered in bulk DNA from blood, saliva and urine, not in any of the iPSC lines. Lineage frequencies in bulk samples (red for blood, blue for saliva and yellow for urine) are shown in logarithmic scale by bar graphs. A new mutation discovered in this study is denoted by Б. From the Fasching*et al.* study (1), the original tree is reproduced/reprinted with permission from AAAS. (**B**) New branches in the tree reconstructed from lines with shallow coverage. New mutations shared by daughter cells are denoted by Cyrillic letters (see Supplementary Table S1). (**C**) Overlap between discovered mosaic mutations from three strategies: original from the Fasching *et al.* study (1), using shallow-coverage data with two strategies: ‘sum all’ and ‘pick one’. (**D**) Sanger validation in RT#13 of a newly discovered indel from recessive lineage (Supplementary Figure S3). Overlapping peaks in the Sanger trace perfectly match to the reference sequence and the sequence with the indel.

Based on all these summed/picked BAM files, we called mosaic SNVs and indels through an exhaustive pairwise comparison of all clusters and high-coverage lines. For each pairwise comparison, one cluster/line was considered as a tumor and another one as a reference normal. We used consensus calls with PASS value made by both Mutect2 (8) and Strelka2 (10) to identify mutations in each pairwise comparison. The exhaustive comparison allowed us to find mutations that are shared by multiple lines, while subsequent application of the All^2^ (https://github.com/abyzovlab/all2) filtering strategy resulted in filtering out false positives to arrive at an accurate mutation call set (11). Specifically, for each mutation, the All^2^ approach works by finding two complementary sets of clusters/lines, namely A and B, from an individual, where clusters/lines in set A will have the mutation, while those in set B will not (11), so that consistent calls for the mutation can be made when comparing clusters/lines from set A to those from set B. Patterns of calls made for the mutation when comparing clusters/lines between A and B sets are used to calculate mosaic and germline scores and to determine whether a call is a mosaic mutation, germline variant or false positive (Figure 1C). This strategy enabled us to identify high-frequency mosaic mutations that occurred during early development and therefore were present in most clusters/lines, thus resembling germline variants, and being missed by most mutation callers when compared to the matched reference bulk. Calls were required to have at least 30% variant allele frequency (VAF) to ensure removal of mutations introduced during culturing clones. Applying higher VAF threshold (40%) eliminated some of the shared mutations, which although not impacting lineage reconstruction in LB may possibly impact lineage reconstruction in other individuals. Applying lower VAF threshold (20%) resulted in calling mutations conflicting with tree branches and likely reflecting false positive calls. Additionally, only indels <10 bp were used to retain most confident calls. All^2^ output contains the list of discovered mosaic SNVs/indels and their sharing between clusters and iPSC lines, which is the necessary information used for the lineage reconstruction (Figure 2A).

All^2^ comparisons across a larger number of cells/lines could be computationally intensive (e.g. for 100 cells/lines, we need to make 100 × 99 = 9900 comparisons). In such cases, the computations can be reduced by restricting comparisons to the sites of called germline-like variant (e.g. with GATK HaplotypeCaller) heterogeneous across compared lines (i.e. called in some but not all cells/lines).

### Sanger validation for a newly discovered indel

Amplicon-seq was performed to validate the newly discovered indel. Forward and reverse primers were designed by selecting a DNA template of 800 nucleotides containing the candidate variant (variant ± 400 bp), with amplicon size of 226 bp. For PCR amplification, we used the Phusion High-Fidelity DNA Polymerase (Thermo Fisher) to minimize the polymerase error rate; optimal annealing temperature was defined using the *T*_m_ calculator tool available on the Thermo Fisher website. Primer specificity was initially confirmed *in silico* with UCSC Genome Browser (http://genome.ucsc.edu/index.html), and then confirmed on gel by the presence of a unique PCR product of the expected size. Amplicon DNA was then purified using the QIAquick PCR Purification Kit (Qiagen) and submitted for Sanger sequencing, together with its forward primer. Forward primer: GAGATCCCACCATTGCACTC; reverse primer: TGCACTTTATCCTCCCACCT.

### Calling mutations in mitochondrial genome

Mitochondria are present in cells in hundreds of copies. Furthermore, their inheritance is not deterministic. Because of these reasons, mutations in mitochondria can be at variable frequencies in cell progenies and even disappear. Therefore, one cannot simply rely on the presence or absence of mutations in mitochondria to reconstruct lineage. Instead, we utilized multiple mutations in mitochondria and their VAF to cluster lines. First, we called mutations in the mitochondrial genome by application of comprehensive pairwise comparison between all 72 iPSC lines utilizing their full coverage (Supplementary Figure S4A). We used Mutect2 (8) to call the SNVs and indels from each pairwise comparison between lines. Inherited variants and likely false positives were filtered using All^2^ tool (11) yielding 126 mutation calls with mosaic score above zero (Supplementary Figure S4B). Additionally, to select higher confidence calls we applied the *χ*^2^ (Benjamini–Hochberg corrected *P*-value = 0, reflecting values below double float point precision) statistical test to retain calls that had heterogeneous frequency across lines and thus are unlikely to be a systematic low-frequency artifact of sequencing or alignment. This resulted in a set of 82 high-confidence mitochondrial mutations (Supplementary Figure S4C).

### Clustering of iPSC lines using mutations in mitochondrial genome

To identify clusters of iPSC lines using the VAF of mitochondrial mutations, we used a hierarchical clustering algorithm with the sample-by-sample distance (1 – *ρ*) matrix, where *ρ* is Pearson’s correlation coefficient of VAFs between samples. Ward’s minimum variance method was used for agglomerating clusters in the same way as we did for the nuclear genome. Two representative hierarchical trees with clusters using two set of mutations, all mutations (Supplementary Figure S4B) and highly confident ones (Supplementary Figure S4C), were constructed.

### Analyzing clonal data for myeloproliferative neoplasms

Data for a study that derived and sequenced clonal hematopoietic colonies from patients with myeloproliferative neoplasms (12) were downloaded from the European Genome–Phenome Archive (https://www.ebi.ac.uk/ega/home) with accession code EGAD00001007714 (WGS colonies). We analyzed data for two patients: PD6629 and PD5117. Since no other samples (besides clonal hematopoietic colonies) were sequenced in that study, we constructed a pseudo-bulk sample simulating (to the extent possible) other normal samples from the same individual. The pseudo-bulk construction was accomplished by combining data for outstanding clones in the trees for selected patients. Specifically, in patient PD6629 we used clones PD6629an, PD6629db_lo0016 and PD6629db_lo0026 because they belong to the outlier clade not harboring *DNMT3A*:p.R882H mutation. In patient PD5117, we used clones PD5117am, PD5117ck, PD5117dc and PD5117s that were chosen randomly out of colonies not harboring the *JAK2*:p.V617F mutation. Data for other clones were downsampled as for iPSC lines (see above). Initial mutations were called relative to constructed pseudo-bulks. Subsequent analyses were conducted as for iPSC lines. Particularly, in patient PD5117 we found a ‘Unique’ cluster (C2 cluster in Supplementary Figure S6B) out of which we picked 12 colonies and then we utilized their full coverage, same as in calling mutations for clusters and high-coverage iPSC lines (see above).

### Statistical analyses

R v4.0.3 was used for statistical analysis. Hierarchical cluster analysis was accomplished by the ‘dist’ and ‘hclust’ functions from the ‘stats’ R package. For distance measure, ‘method = binary’ and ‘method = pearson’ were given to the ‘dist’ function for measuring the dissimilarity of mutation occurrences and VAFs between samples, respectively. For the linkage method, ‘method = ward.D2’ was given to the ‘hclust’ function. *χ*^2^ test with Benjamini–Hochberg correction for multiple testing was performed to test the independence of read count between reference and altered alleles across samples.

### Software availability

The manuscript describes strategy/approach that is based on the application of other tools: SAMtools (7), Mutect2 (8), Strelka2 (10), All^2^ (11) (https://github.com/abyzovlab/all2) and custom R scripts. Scripts used for analysis and processing results are provided in the Supplementary Data.

## RESULTS

### Tracing lineages using shallow-coverage iPSC lines

In a 27-year-old male (termed LB), we previously derived 74 iPSC lines from six biopsies (1). Out of these lines, 25 iPSC lines (prescreened by genotyping for being nonredundant) were sequenced to 30× or higher coverage. In the current study, we tested whether clustering multiple iPSC lines sequenced at low coverage could provide equal discovery power for mutations and lineage reconstruction as sequencing single lines at high coverage. Therefore, we downsampled coverage in each of those 25 lines to 5× and sequenced the remaining 47 lines to a 5× coverage (Supplementary Figure S1). We then applied our discovery pipeline for mosaic mutations in each of the 72 sequenced lines by comparing the iPSC shallow-coverage genomic data to those with high coverage from blood (see the ‘Materials and Methods’ section). Shallow-coverage data allowed discovering roughly half of the mosaic SNVs in each line, as compared to those that were previously found from high-coverage data (Figure 1A). Since every iPSC line has 1000–2000 mosaic SNVs inherited from the founder fibroblast cells, discovering half of them from 5× data was sufficient to cluster iPSC lines (Figure 1B). We also estimated that a mutation burden of ∼100 mutations per line would allow us to produce the same clusters as with the original mutation burden (see the ‘Materials and Methods’ section). In most clusters, iPSC lines were derived from the same biopsy, consistent with clusters representing lineages from local clonal expansion. In support of that, those clusters typically contained just one iPSC line previously selected as not redundant based on our previous genotyping screen and sequenced at high coverage (1). One outlier cluster, termed ‘Unique’, included multiple nonredundant iPSC lines (Figure 1B, starred), and thus contained lines that represent unique lineages. Because these lineages are represented by a single iPSC line, using them for lineage reconstruction requires each one to be sequenced at high coverage.

### Reconstructing the lineage tree during development

We applied a ‘sum all’ strategy for lineage reconstruction. Specifically, from ‘Unique’ clusters we picked only those 10 lines that were previously sequenced to high coverage and utilized their full coverage (Figure 1B, starred). For other clusters, we summed shallow-coverage data from all lines in each cluster, resulting in 13 clusters with typical coverage of 15× or higher. By systematically comparing 23 selected clusters and lines using the All^2^ approach (see the ‘Materials and Methods’ section), we identified a total of 27 891 mosaic mutations (Figure 1C). The distribution of mutation calls by mosaic and germline scores was similar to the one observed in the original discovery using high-coverage data for the 25 iPSC lines (11). Using the discovered mutations and their sharing between clusters and iPSC lines, we almost perfectly replicated the original lineage tree (Figure 2A). Of 71 mutations listed in the original tree, two (termed w and q′) were not called and one (termed l′) did not pass post-calling filtering. Since branches can have multiple mutations, only one branch in the original tree (marked by q′ and grayed) was not replicated. However, we reconstructed two additional branches in the lineage originally represented by the LA1#3 line (Figure 2B). Two clusters in that lineage, LTC3 and LTC4, consisted of iPSC lines that had not been sequenced in the previous study and thus the corresponding branches could not be discovered.

Some clusters can be rather small, e.g. clusters RTC3 and LTC4 have only two lines, and their combined coverage may not be high enough to ensure accurate discovery of all mutations. To mitigate this issue, we envisioned an enhanced strategy for mutation discovery and tree reconstruction by randomly picking one iPSC line per each small cluster and sequencing it to high coverage (‘pick one’ strategy). To test the efficacy of this strategy while capitalizing on existing data, we randomly picked an iPSC line with high-coverage sequencing data for each small cluster (i.e. for LA2C2, RA1C2, LTC2 and RTC3). By systematically comparing 21 lines/clusters, we missed only one early mutation, w (Figure 2C), suggesting that the ‘pick one’ strategy is slightly more accurate than the ‘sum all’. A slight drawback of the ‘pick one’ strategy is that it requires generation of additional high-coverage data and thus is slightly more expensive.

Small differences in the number of discovered early mutations between strategies are likely driven by stochasticity in sampled lines and utilized data, suggesting that discovery of lineage marker mutations from shallow coverage has the same power as from high-coverage data. There was a larger discordance between strategies for the total number of discovered mutations, but that is driven by the different power to discover later mutations that are present at smaller frequencies (Supplementary Figure S2). In support of this, using the low-coverage strategy we discovered an additional indel in three lines (LA2#2, LT#3 and RA2#54). Additional analysis revealed that it is likely present in all iPSC lines from the recessive lineage and absent in all iPSC lines from the dominant lineage, i.e. is consistent with the tree (Supplementary Figure S3). This inference was validated by Sanger sequencing of the locus with the indel in several iPSC lines from dominant and recessive lineages (Figure 2D and Supplementary Figure S3). Thus, this indel is a bona fide marker mutation for the recessive lineage. The indel results in an addition of adenine in the poly-A sequence, which is hard to ascertain due to high mutability and elevated sequencing errors mimicking indels. This likely explains why it was missed in the previous analysis and was discovered only in a few lines in the described analysis.

### Lineage tree reconstruction using mutations in mitochondrial genome

In every cell, there are two copies of the nuclear genome (except for sex chromosomes) but hundreds and thousands of copies of the mitochondrial genome. Accordingly, during sequencing, coverage of the mitochondrial genome is hundreds to thousands times higher than that of the nuclear genome, enabling comprehensive ascertainment of the mitochondrial genome even if coverage is shallow. We investigated whether mutations in the mitochondrial genome could be used for precise lineage reconstructions. In LB, we discovered a total of 126 mutations in the mitochondrial genome across all iPSC lines (see the ‘Materials and Methods’ section). Clustering of iPSC lines based on these mutations mostly reproduced clusters based on mutations from the nuclear genome; however, a dozen lines were clustered incorrectly (Supplementary Figure S4). For example, lines from cluster RA2C1 were consistently not clustered together because they lacked unique mutations in their mitochondria. Similarly, several lines from the ‘Unique’ cluster were spread in other clusters (Supplementary Figure S4B and C). Some other clusters were only partially reproduced because of infusion of lines from other clusters. We concluded that using mutations in the mitochondrial genome is not optimal for a precise reconstruction of lineage trees.

### Assessment of iPSC line clonality from shallow-coverage data

**Figure 3.**
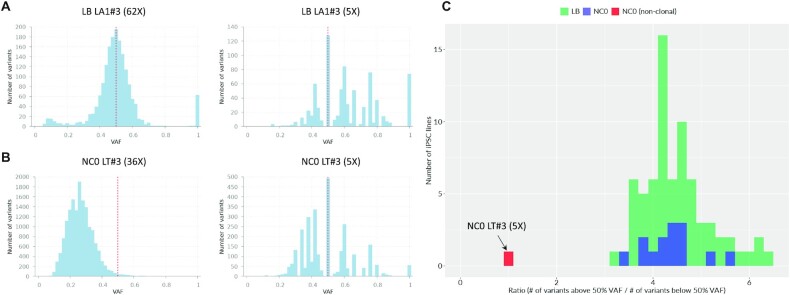
Quality control for clonality of iPSC lines from shallow-coverage WGS data. (**A**) VAF distribution of discovered mutations for a clonal iPSC line from LB individual. (**B**) VAF distribution of discovered mutations for a nonclonal iPSC line from NC0 individual. (**C**) Distribution of the ratio of mutations with high (over 50%) and low (below 50%) VAFs from 5× coverage data for clonal (green for LB individual and blue for NC0 individual) and nonclonal (red in only NC0) iPSC lines.

While most of the iPSC lines are clonal, occasionally some are derived from two or more cells (1). From high-coverage data, such lines can be identified from the VAF distribution of mosaic mutations in the line. In clonal lines, the VAF distribution looks like a symmetrical bell-shaped curve centered around 50%, i.e. almost all mutations are on one haplotype of autosomes. Such mutations are from the founder cell of an iPSC line and thus inherited by all cells in the clone (Figure 3A). There could be mutations that arose during culture and have smaller VAF, but they are a minority. For nonclonal lines, the VAF of mutations discovered on autosomes is below 50% (Figure 3B). Inferring precise VAF from shallow-coverage data is problematic (Figure 3A and B). However, we capitalized on the fact that discovering low-frequency mutations is less sensitive than discovering high-frequency mutations. Empirically, we observed that from shallow-coverage data the ratio of discovered high-frequency (>50% VAF) and low-frequency (<50% VAF) mutations is different for clonal and nonclonal lines (Supplementary Figure S5), allowing for the nonambiguous distinction between such lines (Figure 3C). We suggest that ratios above 3 are indicative of clonal iPSC lines, while ratios below 2 are indicative of nonclonal lines. Ratios between 2 and 3 are inconclusive to determine clonality of a line.

### Lineage tree reconstruction in cancers

**Figure 4.**
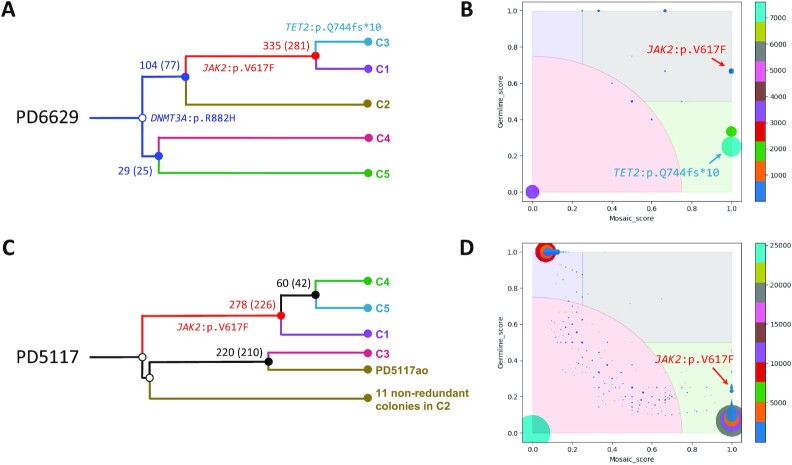
Lineage tree reconstruction from WGS data from clonal hematopoietic colonies in myeloproliferative neoplasms (12). (**A**) Reconstructed lineage tree for patient PD6629. The tips of the branches represent the clusters of colonies. Each of them is colored in the same color as the corresponding cluster in Supplementary Figure S6A. Each shared branch is annotated with the number of shared mutations across downstream descendants. Approximate mutation count inferred from graphical lineage trees in the original study (12) is shown in parentheses. Shared branches containing cancer driver mutations are highlighted by different colors. (**B**) The distribution of discovered mutations in patient PD6629 by mosaic and germline scores from All^2^ comparison. Likely mosaic mutations are located in the green and gray areas, likely germline variants are in blue area and false calls are in red area. Scores for driver mutations are pointed out by arrow. (**C**) Reconstructed lineage tree for patient PD5117. Colors of clusters are same as in Supplementary Figure S6B. (**D**) Mosaic and germline score distribution of discovered mutations in patient PD5117. Colors are as in panel (B).

To demonstrate the applicability of this approach to study the progression of cancer, we used publicly available data used to reconstruct lineages in myeloproliferative neoplasms (12). We analyzed data for clonal hematopoietic colonies from two patients: PD6629 and PD5117. Since apart from colonies no other samples were sequenced in that study, we used some clones to construct pseudo-bulk sample to enable comparison for discovering mutations in shallow-coverage data for other clones. Several clones did not pass the check for clonality and were excluded from the analysis (Supplementary Figures S7 and S8). For all other clones, we conducted lineage reconstruction analysis the same way we did for iPSC lines above. In both patients, we identified five clusters of clones (Supplementary Figure S6) and then combined WGS data within the clusters and picked 12 nonredundant clones from the ‘Unique’ cluster (Supplementary Figure S6B) in patient PD5117 and used their full coverage. Mosaic mutation discovery was performed in the clusters/lines that allowed to infer clonal history of these myeloproliferative neoplasms (Figure 4). In each patient, and in agreement with the original report, we discovered a cluster(s) corresponding to the neoplasm along with the corresponding driving mutations in *JAK2*, *DNMT3A* and *TET2* genes.

## DISCUSSION

The analysis presented demonstrates how shallow-coverage sequencing data can be efficiently used to reconstruct cell lineages from clonal cell lines. We propose the following seven-step strategy for lineage reconstruction: (i) derive multiple lines (or clones) from biopsies/regions from a biological system of interest (e.g. body or cancer); (ii) sequence all lines at shallow 5× coverage (this can be done as soon as lines become available); (iii) discover mutations in each line relative to some bulk normal tissue (e.g. blood) and eliminate nonclonal lines; (iv) cluster the lines based on the discovered mutations; (v) define clusters with redundant lines and combine data for lines in each redundant cluster (at this step, a draft lineage tree can already be generated); (vi) sequence unique lines (i.e. lines that do not share mutations with other lines and therefore cannot be clustered) to a coverage of 15× to 30×; and (vii) discover mutations in the clusters and unique lines and reconstruct the lineage tree. The described strategy is cost-efficient if compared to sequencing all derived clones at high coverage. For the reconstruction of lineage tree in LB, using the proposed strategy as opposed to sequencing all iPSC lines would have saved ∼$26 000, assuming a cost of $600 for 30× WGS and a cost of $150 for 5× WGS. The strategy is on par in cost but time-efficient if compared to our original strategy when iPSC lines were prescreened for marker mutations of early lineages (1). Because of the necessity of deriving a draft lineage tree and the need to genotype most of the iPSC lines to define mutations that mark early lineages, the original strategy takes at least two extra months and adds some extra cost (for amplicon-seq genotyping) to the experiment.

The main underlying assumption of the strategy proposed here is that there is a high chance of sampling redundant clones/lines. This assumption is true for fibroblasts derived from skin biopsies (1,13) but may not be universally true for all tissues or biopsy sites in the human body. For example, cells in the blood are continuously mixed and one might expect that random sampling of cells for cloning or derivation of iPSC lines can result in mostly nonredundant lines. However, in aging individuals, where clonal hematopoiesis is highly prevalent (14), one would likely have a high degree of clone redundancy and the proposed strategy would be applicable, as it is applicable for analyzing clones in hematologic malignancies. Another essential reliance of the approach is that one can detect a sufficient count of mutations from shallow coverage to find redundant clones. Cells of a newborn human have hundreds and up to a thousand mutations (13,15), such that several hundred mutations per cell can be discovered at shallow coverage, rendering the strategy applicable to lineage reconstruction. However, the strategy may not be that efficient when dealing with fetal cells with low mutation burden. It is also worth noting that merging 5× WGS data limits the discovery of mutations that are private to each of the cell line/clone.

Further reduction in the sequencing coverage of iPSC lines is unlikely to give a major decrease in experimental cost. Slight reduction of shallow coverage (e.g. to 4×) is unlikely to affect clustering of redundant lines but may require additional sequencing of lines from clusters with just two or three lines. Sequencing at 1× or 2× coverage, even if resulting in correct clustering of redundant lines (large number of mutations per cell will be crucial), would likely require sequencing to high coverage of some lines from each cluster. Since at shallow coverage most of the sequencing cost is taken by library preparation, sequencing at 1–2× would only be beneficial if the same sequencing library can be reused, which is challenging to achieve in practice. Thus, beyond the strategy outlined here, it seems likely that one of the major next advances leading to further improvement in cost efficiency in lineage tracing would come from reduction in the cost of library preparation for WGS.

## DATA AVAILABILITY

All data presented in our work are included in the text and supplementary material. The primary data are accessible at NDAR (study no. 1057; https://nda.nih.gov/study.html?id=1057) for qualified researchers.

## Supplementary Material

gkad254_Supplemental_Files
