# Supplementary material for: Efficient reconstruction of cell lineage trees for cell ancestry and cancer
Source: Nucleic Acids Res. 2023 Apr 7;51(10):e57. doi: 10.1093/nar/gkad254 (PMC10250207; doi:10.1093/nar/gkad254)
Supplement: gkad254_Supplemental_Files [file gkad254_supplemental_files.zip › Supplementary.pdf]

Supplementary Materials for

Efficient reconstruction of cell lineage trees for cell ancestry and cancer

Yeongjun Jang†, Liana Fasching†, Taejeong Bae, Livia Tomasini, Jeremy Schreiner, Anna Szekely, Thomas V. Fernandez, James F. Leckman, Flora M. Vaccarino*, Alexej Abyzov*

*Corresponding author. Email: abyzov.alexej@mayo.edu (A.A.) and flora.vaccarino@yale.edu (F.M.V.)

This file includes:

Supplementary Figures S1 to S9
Supplementary Table S1

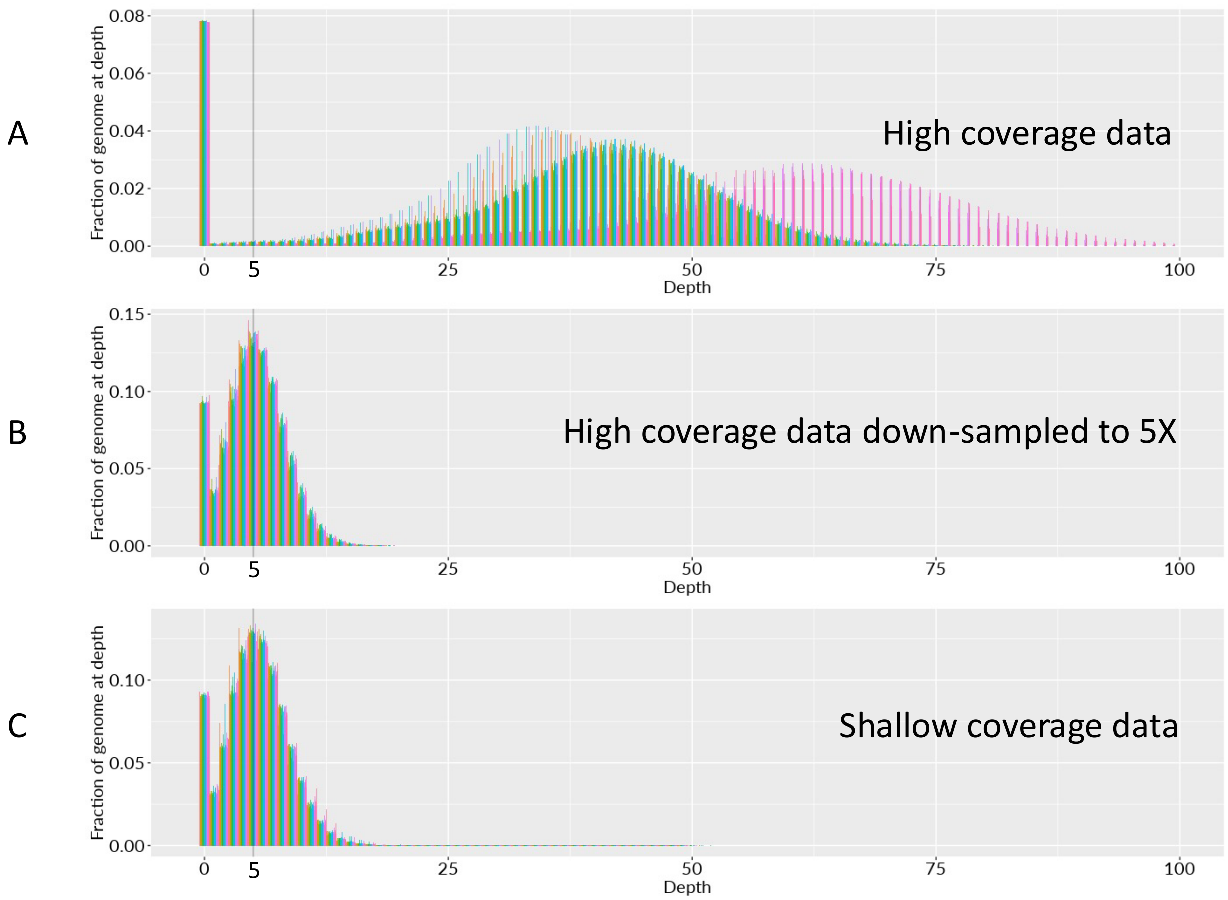

Figure S1. Coverage distribution of high, shallow coverage and down-sampled iPSC lines. **(A)** Coverage distribution for 25 high coverage iPSC lines. Distribution for each line is shown by a separate color. **(B)** Coverage distribution for 25 high coverage iPSC lines after down sampling to ~5X. Distribution for each line is shown by a separate color. **(C)** Coverage distribution for 47 low coverage iPSC lines. Distribution for each line is shown by a separate color.

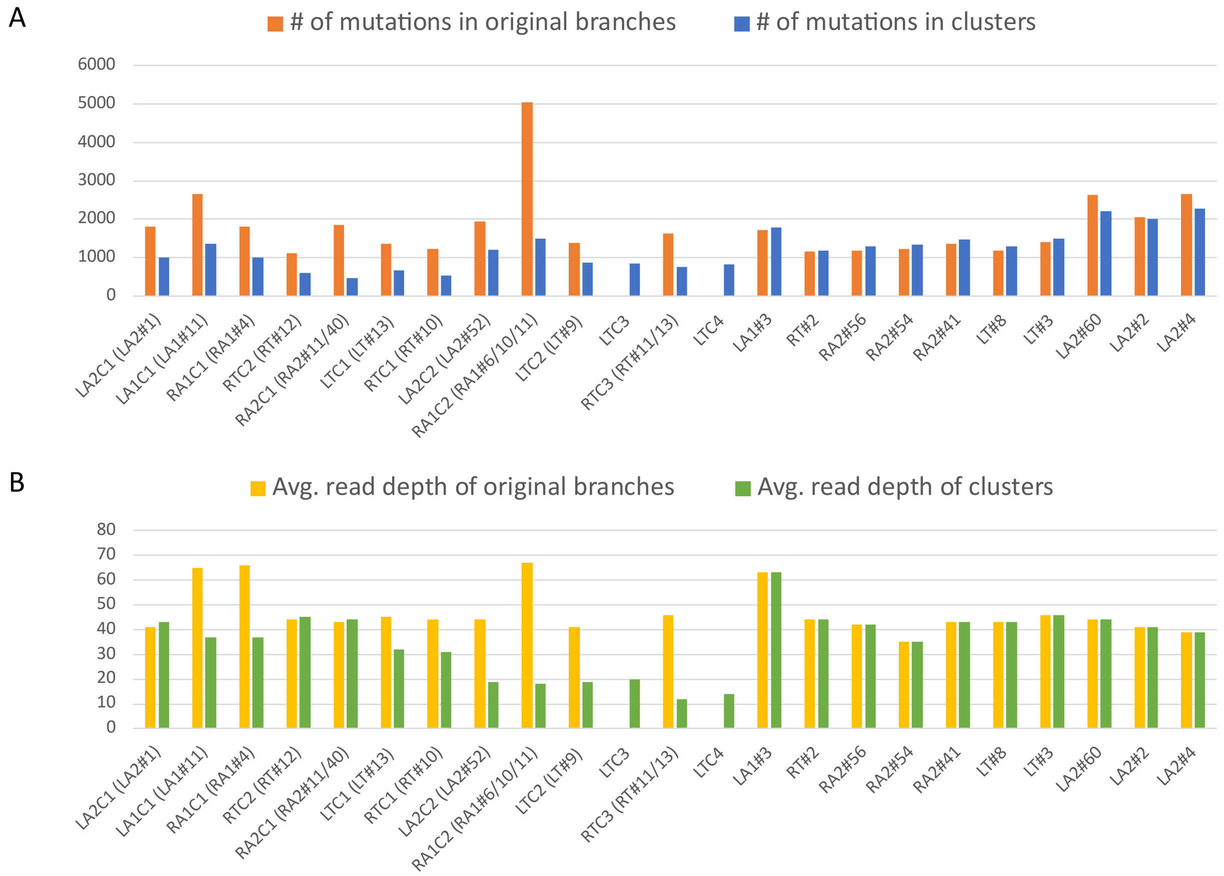

Figure S2. Comparison of the number of mutations and coverage between clusters of iPSC lines (Fig. 1B) and branches in the original tree (*1*).

Each category is named by a cluster followed in parenthesis by the names of high coverage iPSC lines in the same branch. **(A)** Number of mutations found in original branches (orange bars) discovered from high coverage sequencing of the indicated iPSC lines and their matched clusters (blue bars) discovered from combining shallow sequenced iPSC lines. More mutations are discovered from high coverage sequenced lines. This is because when combining sequence data for shallow coverage lines in the cluster, only earlier, shared mutations will have high frequency in the combined coverage and can be called for a cluster, while later mutations will not be called. **(B)** Average read depth of original branches (yellow bars) and clusters (green bars).

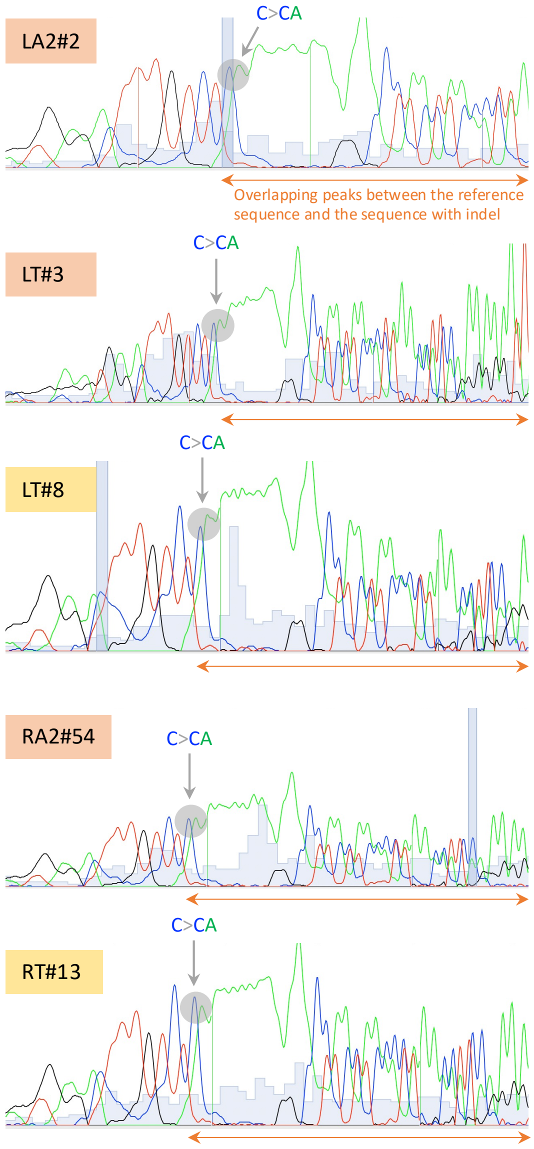

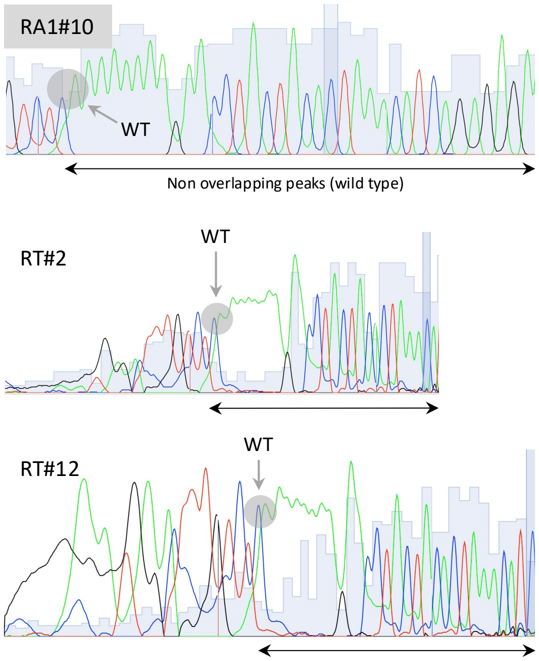

Sample	REF reads	ALT reads	VAF
LA2#2	36	21	0.583
LA2#60	33	12	0.363
RA1#4	73	33	0.452
RA1#10	67	1	0.015
RA2#54	40	21	0.525
LT#3	38	13	0.342
LT#8	30	11	0.367
RT#11	48	11	0.229
RT#13	48	19	0.396

iPSC lines from recessive lineage where the indel is discovered

iPSC lines from recessive lineage where the indel has supporting reads

iPSC lines from dominant lineage where the indel has supporting reads

Figure S3. Validation of a novel discovered indel.

Sanger traces validate the indel in 5 lines of the recessive lineage and invalidate it in 3 iPSC lines of the dominant lineage. The table shows read support for the indel across all iPSC lines. The indel is consistently supported by tens of reads in each iPSC lines from the recessive lineage. Only one iPSC lines (i.e., RA1#10) from the dominant lineage has one supportive read for the indel.

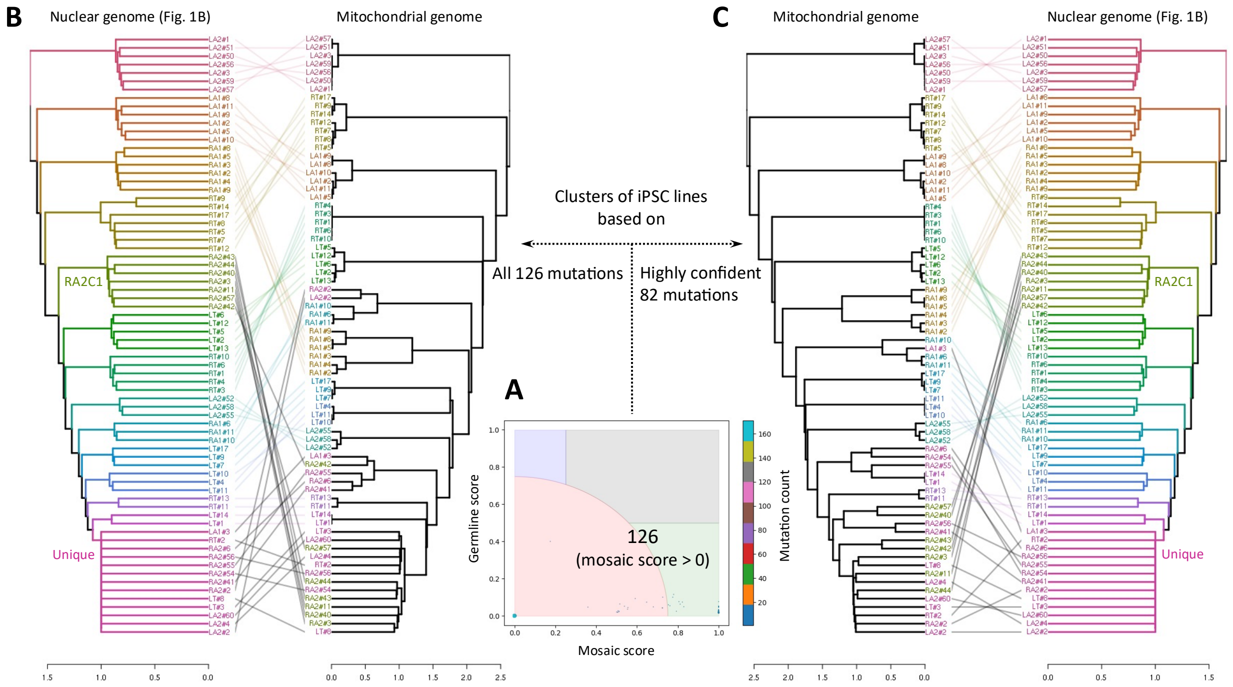

Figure S4. Lineage tree reconstruction from mutations in mitochondrial genome.

(A) Distribution of scores for mutation calls in mitochondria from comprehensive pairwise comparison across 72 iPSC lines. Each dot is a call for a mutation. There are 126 mutations calls with mosaic score above zero. Comparison of hierarchical clustering trees based on the sets of **(B)** all 126 and **(C)** highly confident 82 mutations in mitochondrial genomes and based on mutations in the nuclear genome from 5X coverage data (Fig. 1B). Each cluster of iPSC lines is shown by a separate color. Connections between the nuclear and mitochondrial genomes of each iPSC line are shown by lines colored by the cluster it belongs to. Those iPSC lines that did not reproduce their clusters based on mutations in nuclear genome are denoted by grey connecting lines (i.e., iPSC lines in the “RA2C1” or “Unique”).

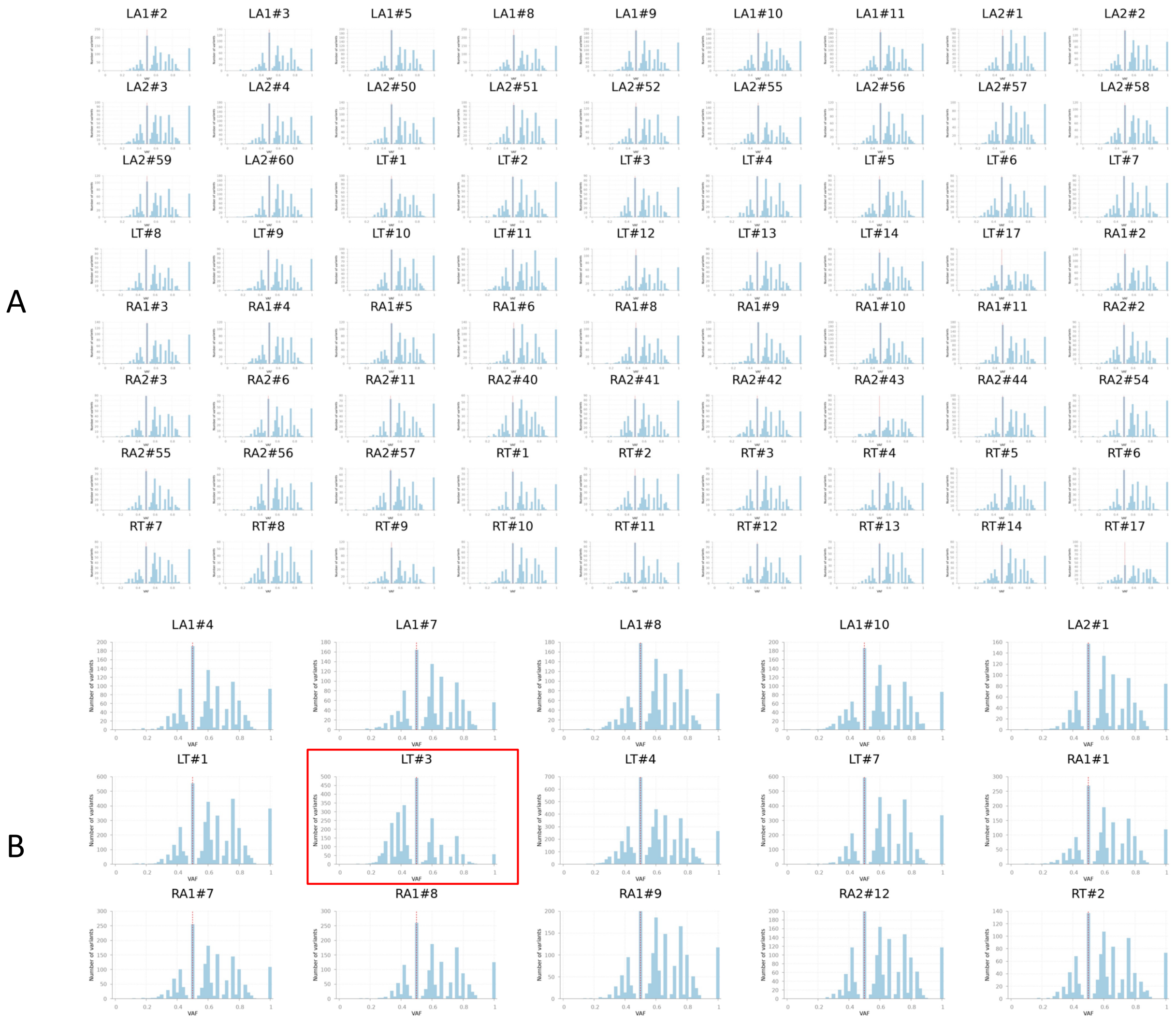
A
LA1#2
LA1#3
LA1#5
LA1#8
LA1#9
LA1#10
LA1#11
LA2#1
LA2#2
LA2#3
LA2#4
LA2#50
LA2#51
LA2#52
LA2#55
LA2#56
LA2#57
LA2#58
LA2#59
LA2#60
LT#1
LT#2
LT#3
LT#4
LT#5
LT#6
LT#7
LT#8
LT#9
LT#10
LT#11
LT#12
LT#13
LT#14
LT#17
RA1#2
RA1#3
RA1#4
RA1#5
RA1#6
RA1#8
RA1#9
RA1#10
RA1#11
RA2#2
RA2#3
RA2#6
RA2#11
RA2#40
RA2#41
RA2#42
RA2#43
RA2#44
RA2#54
RA2#55
RA2#56
RA2#57
RT#1
RT#2
RT#3
RT#4
RT#5
RT#6
RT#7
RT#8
RT#9
RT#10
RT#11
RT#12
RT#13
RT#14
RT#17
B
LA1#4
LA1#7
LA1#8
LA1#10
LA2#1
LT#1
LT#3
LT#4
LT#7
RA1#1
RA1#7
RA1#8
RA1#9
RA2#12
RT#2
Number of variants
VAF

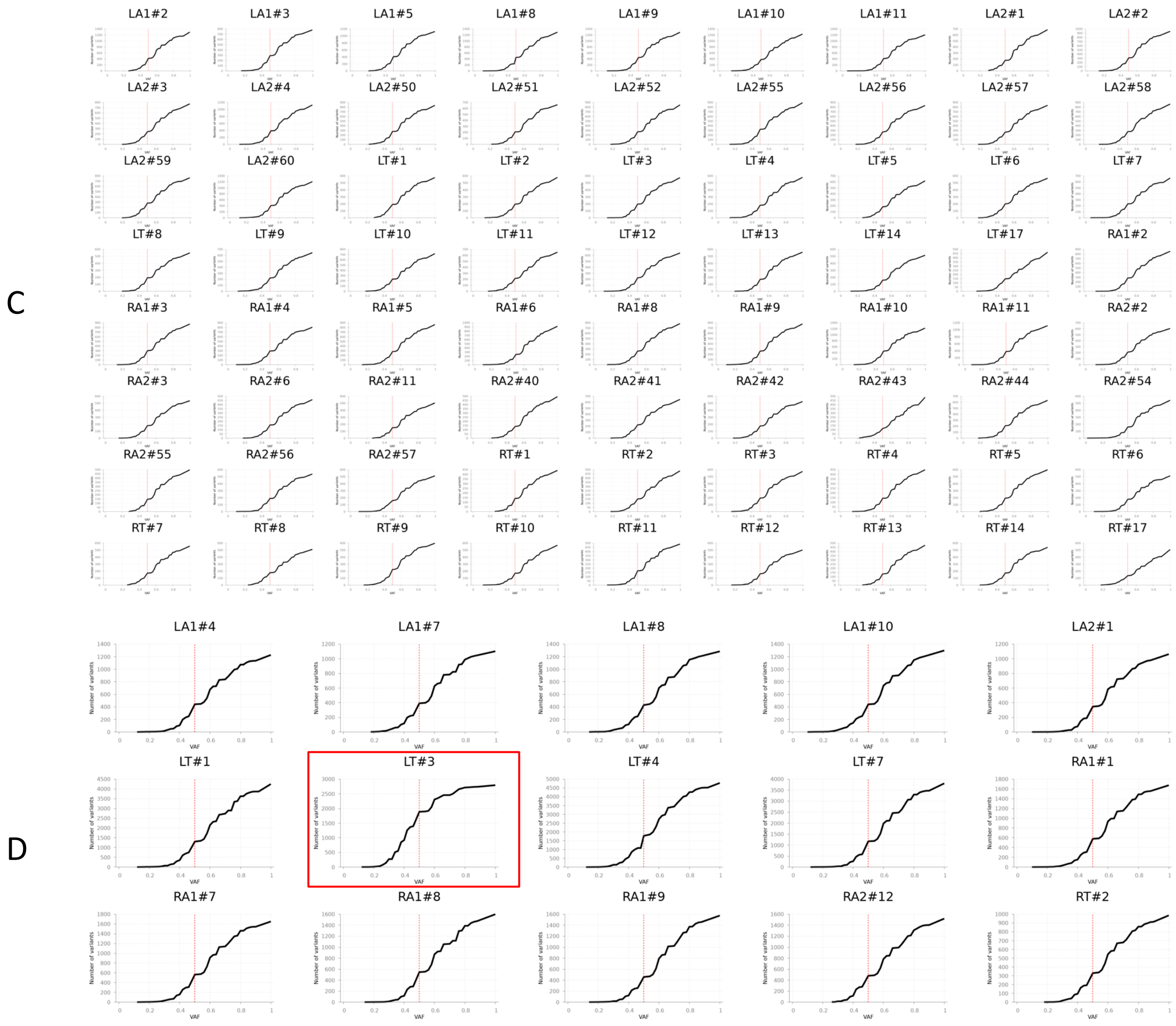

Figure S5. VAF distribution of somatic mutations discovered from 5X coverage data in iPSC lines from 2 individuals.

(A) VAF distribution in iPSC lines from LB individual. **(B)** VAF distribution in iPSC lines from NC0 individual. The distribution for a non-clonal line is highlighted with red square. **(C, D)** The cumulative number of mutations (y-axis) is plotted versus their VAFs (x-axis) for each iPSC line for LB in (C) and NC0 in (D).

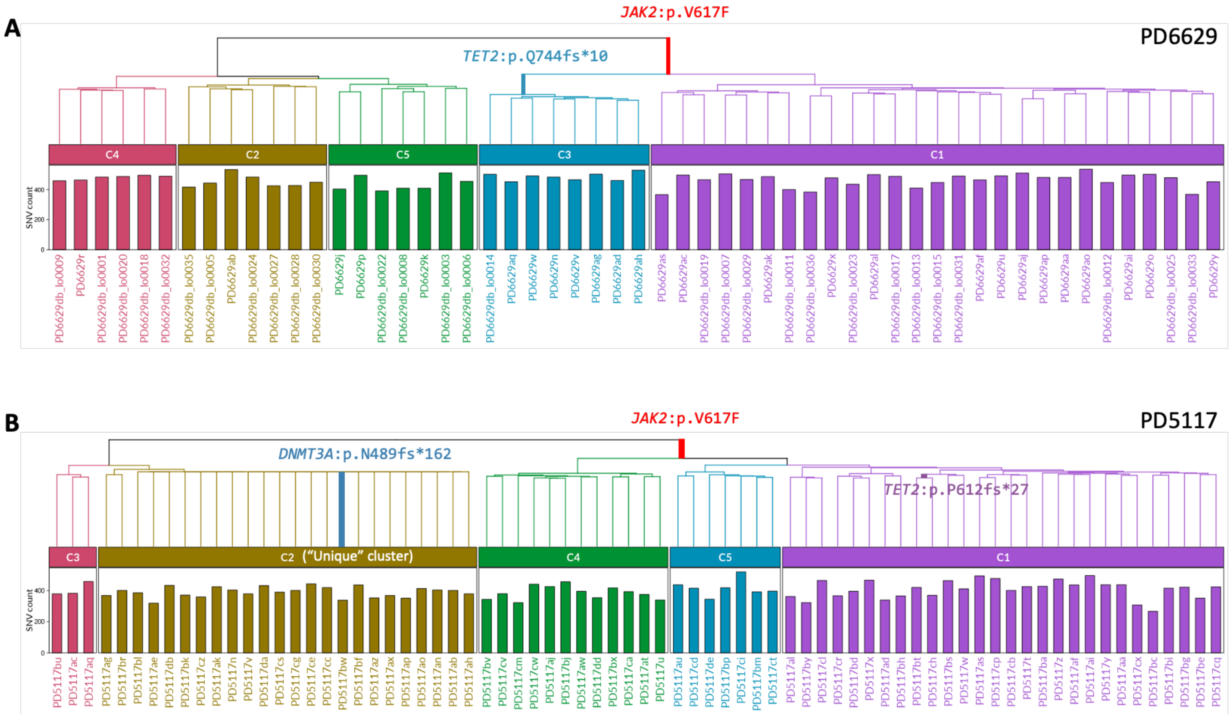

Figure S6. Hierarchical clustering of clonal haematopoietic colonies based on shared mutations discovered from 5X coverage data for two patients with myeloproliferative neoplasm.

(A) Clusters of haematopoietic clones for patient PD6629. Bar plot at the bottom shows counts of discovered somatic SNVs in each clone. Shared branches containing known cancer driver mutations are annotated and highlighted by different colors. **(B)** Clusters of haematopoietic clones for patient PD5117. Colors are as in panel A.

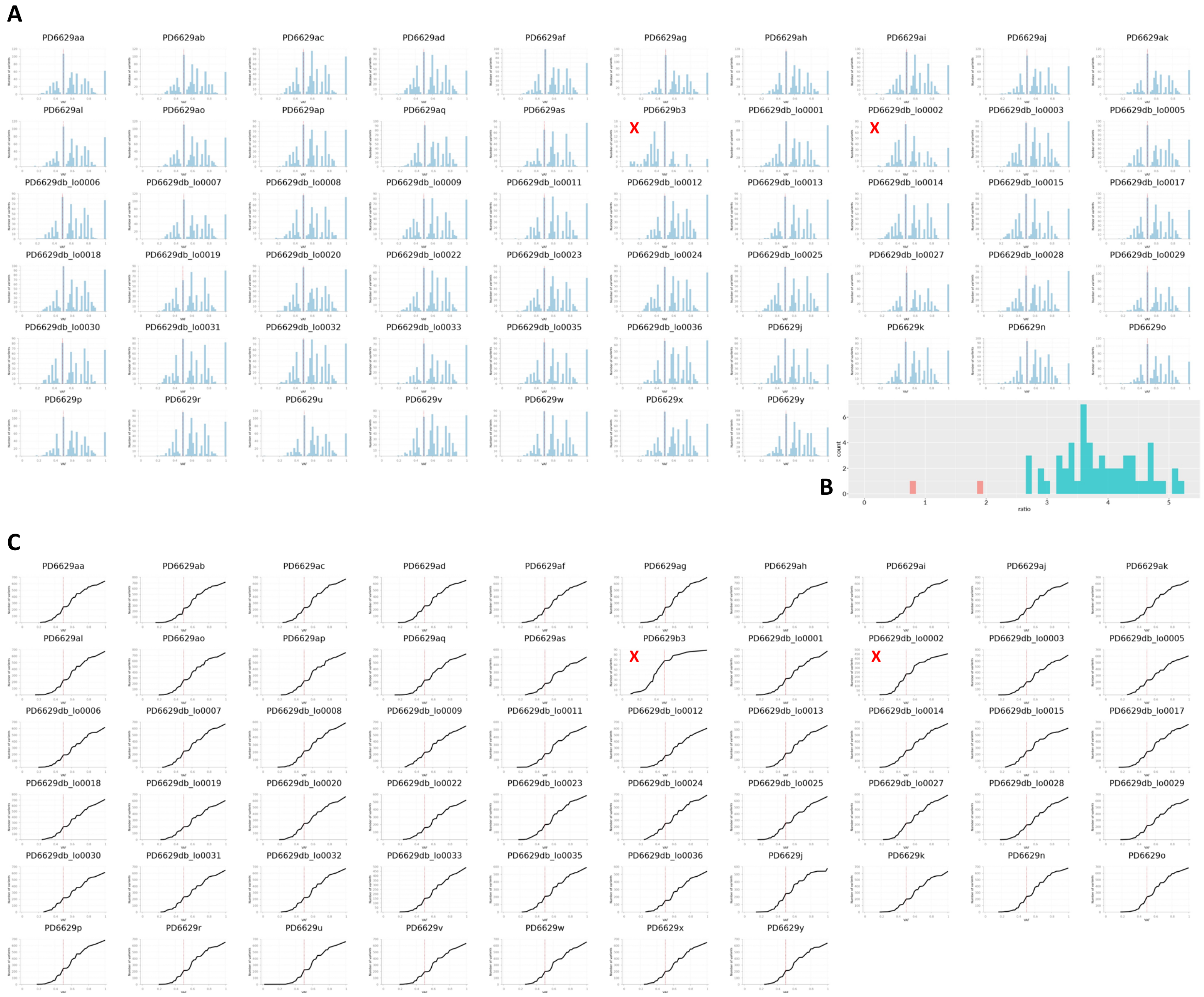

Figure S7. VAF distribution of mutations discovered from 5X coverage data in haematopoietic clones from patient PD6629 with myeloproliferative neoplasm.

(A) VAF distributions in haematopoietic clones from patient PD6629. The distributions for samples that did not pass the clonality check and were excluded from the downstream analyses are marked by "X" in red. **(B)** Distribution of the ratio of mutations with higher (over 50%) VAF over those with lower (below 50%) VAF from 5X coverage data for clonal (in cyan; ratio > 2.5) and non-clonal (in red; ratio ≤ 2.5) samples. Non-clonal samples are those that are marked by "X" in panel A. **(C)** The cumulative number of mutations (y-axis) is plotted versus their VAFs (x-axis) for each clone.

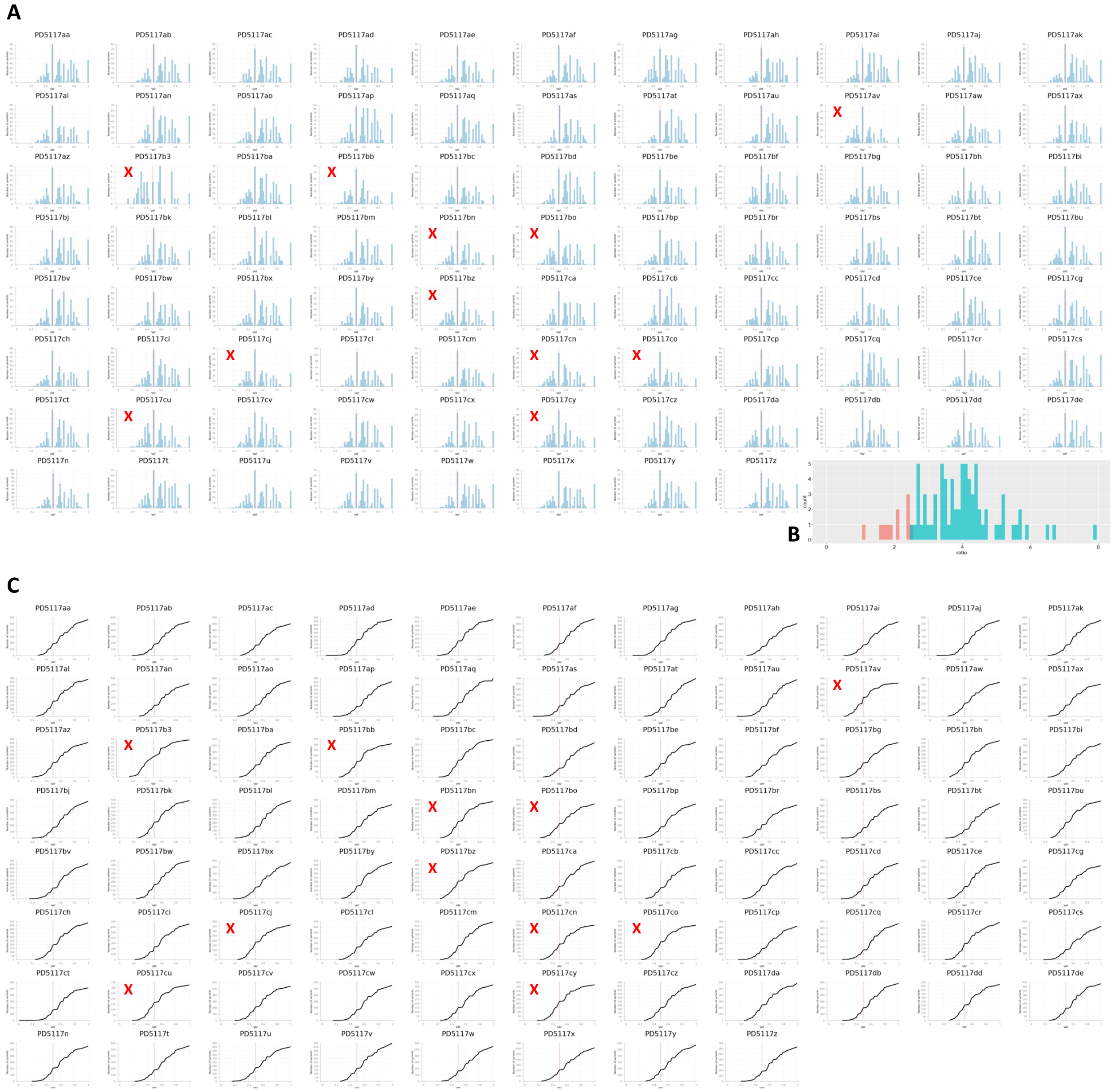

Figure S8. VAF distribution of mutations discovered from 5X coverage data in haematopoietic clones from patient PD5117 with myeloproliferative neoplasm.

(A) VAF distributions in haematopoietic clones from patient PD5117. The distributions for samples that did not pass the clonality check and were excluded from the downstream analyses are marked by "X" in red. **(B)** Distribution of the ratio of mutations with higher (over 50%) VAF over those with lower (below 50%) VAF from 5X coverage data for clonal (in cyan; ratio > 2.5) and non-clonal (in red; ratio ≤ 2.5) samples. Non-clonal samples are those that are marked by "X" in panel A. **(C)** The cumulative number of mutations (y-axis) is plotted versus their VAFs (x-axis) for each clone.

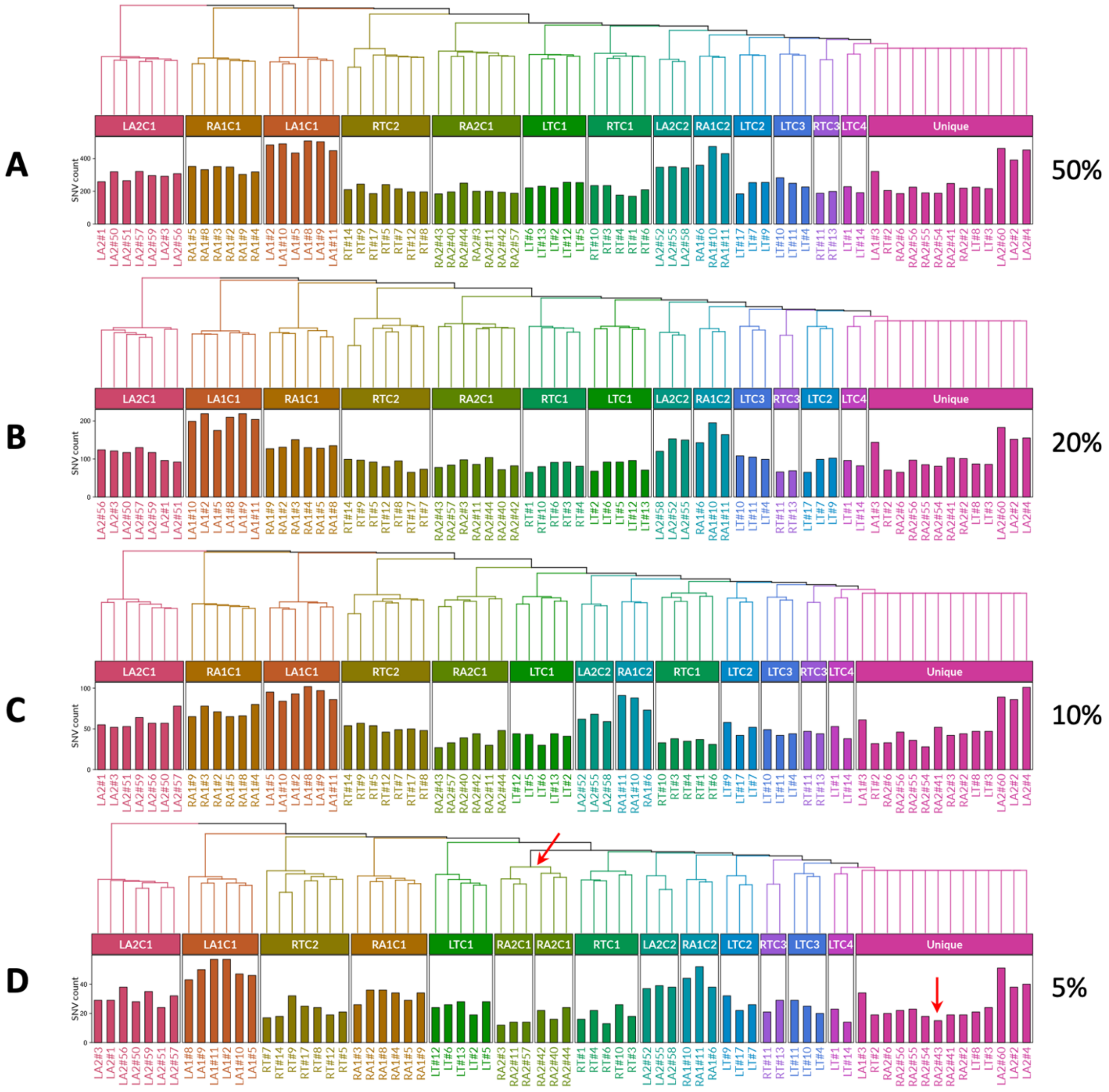

Figure S9. Hierarchical clustering of 5X coverage iPSC lines after simulating lower (50%, 20%, 10%, and 5%) mutation burden in each line.

(A-C) Produced clusters of lines when using random 50% (**A**), 20% (**B**), and 10% (**C**) of all discovered mutations (Figure 1A; roughly 500-1,000 mutations in each line). Bar plots at the bottom of each tree show counts of randomly sampled SNVs in each line. **(D)** Produced clusters of lines when using random 5% (roughly 20-50) of all discovered mutations. Those clusters have discrepancies (highlighted by arrows in red; e.g., RA2C1) as compared to the original clustering obtained using all discovered mutations (Figure 1B).

Table S1. New mutations discovered using shallow coverage data

Two additional branches are reconstructed by these mutations. The full version of this table with chromosomes and positions of mutations will be available in NDA together with primary data.

CHR	POS	REF	ALT	Symbol	Clusters or samples with mutation	VAF in Blood	VAF in Saliva	VAF in Urine
deidentified	deidentified	C	CA	Б	LA2#2, LA2#60, RA1#4, RA1#10, RA2#54, LT#3, LT#8, RT#11, RT#13	2.6%	5.5%	10.5%
deidentified	deidentified	G	A	Ж	LTC3, LTC4, LA1#3	0.9%	0.0%	0.5%
deidentified	deidentified	G	A	Й	LTC3, LTC4, LA1#3	0.7%	0.4%	1.1%
deidentified	deidentified	T	C	Л	LTC3, LTC4, LA1#3	0.0%	0.0%	0.4%
deidentified	deidentified	T	C	Ю	LTC3, LA1#3	1.3%	1.0%	0.0%
deidentified	deidentified	C	T	Я	LTC3, LA1#3	0.5%	0.4%	0.0%
